# Upcycling potato peel waste – Data of the pre-screening of the acid-catalyzed liquefaction

**DOI:** 10.1016/j.dib.2016.04.032

**Published:** 2016-04-21

**Authors:** Patrícia Ventura, João Carlos Moura Bordado, Maria Margarida Mateus, Rui Galhano dos Santos

**Affiliations:** CERENA, Departamento de Engenharia Química e Biológica, Torre Sul, Instituto Superior Técnico, Av. Rovisco Pais, 1049-001 Lisboa, Portugal

## Abstract

Herein, the data acquired regarding the preliminary and exploratory experiments conducted with potato peel as a biomass source for the direct thermochemical liquefaction is disclosed. The procedure was carried out in a 2-ethylhexanol/DEG solvent mixture at 160 °C in the presence of p-Toluenesulfonic acid. The adopted procedure afforded a bio-oil in high yield (up to 93%) after only 30 min. For longer reaction times, higher amounts of solid residues were obtained leading, consequently, to lower yields.

**Specifications Table**

**Table:** 

Subject area	*Chemistry*
More specific subject area	*Chemical Engineering*
Type of data	*Figure*
How data was acquired	*Conversion yield estimated based on solid residue content*
Data format	*Analyzed*
Experimental factors	*Potato peel samples were subjected to moderate temperature in the presence of an acid catalyst and polyhydric alcohols without any pre-treatment whatsoever*
Experimental features	*Thermochemical liquefaction of cork catalyzed by acids*
Data source location	*Lisbon, Portugal, GPS*: 38° 44′ 10.31″N; 9° 08′ 19.66″W
Data accessibility	*Data is provided in the article*

**Value of the data**

• The data set henceforth disclosed regards the first direct thermochemical liquefaction of potato peel.
• The procedure can represent a solution for the mitigation of industrial waste.
• The results presented indicates that this residue can be liquefied leading to a bio-oil which can refined into valuable chemicals and bio-fuels.

## 1 Data

The data provided in this short communication regards the preliminary liquefaction experiments conducted with potato peel in polyhydric alcohols catalysed by p-toluenesulfonic acid with high yield, which, to the best as we know, has never been disclosed.

## 2 Experimental design, materials and methods

Potatoes (*Solanum* *tuberosum* L.) were bought in a local market. The chemical grade reagents and solvents used were acquired from Sigma-Aldrich.

### 2.1 Liquefaction procedure

The method for the liquefaction reaction was adopted from the work previously described [1], [2], [3], [4], [5]. The reaction vessels were loaded with the solvent mixture [1/2 w/w ratio of 2-ethylhexanol and diethylene glycol (DEG)], containing a 3% of p-Toluenesulfonic acid (p-TsOH) and 10% w/w of potato peel. The reaction vessels were then heated to 160 °C, for the desired time. Then, the vessels were allowed to cool to room temperature for further analysis.

### 2.2 Measurement of liquefaction extent

The conversion was gravimetrically evaluated based on the residue content (unreacted raw material). The reaction mixture was diluted with acetone and filtered afterwards the residual solid was washed with acetone and then dried in an oven set at 120 °C until constant weight. The liquefaction yield was calculated by Eq. (1).

(1) $$\mathrm{Liquefaction}\;yi\mathrm{eld}\left(\%\right)=\left(1-\frac{M_{2}}{M_{1}}\right)\times 100$$

where *M*_1_ is the initial mass of cork, *M*_2_ the mass of the residue obtained.

## 3 Data analysis

**Fig. 1:**
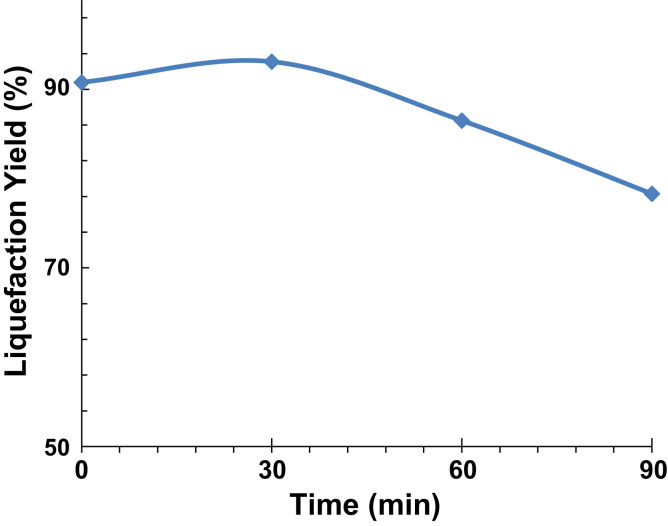
Liquefaction of potato peel at 160 °C in 2-ethylhexanol/DEG.

The data obtained is schemed in Fig. 1.
